# Novel Therapeutic Effects of Non-thermal atmospheric pressure plasma for Muscle Regeneration and Differentiation

**DOI:** 10.1038/srep28829

**Published:** 2016-06-28

**Authors:** Jae Won Choi, Sung Un Kang, Yang Eun Kim, Ju Kyeong Park, Sang Sik Yang, Yeon Soo Kim, Yun Sang Lee, Yuijina Lee, Chul-Ho Kim

**Affiliations:** 1 Department of Otolaryngology, School of Medicine, Ajou University, Suwon, Republic of Korea; 2 Department of Molecular Science & Technology, Ajou University, Suwon, Republic of Korea; 3 Department of Electrical and Computer Engineering, Ajou University, Republic of Korea; 4 ICD Co., Ltd. Anseong, Republic of Korea

## Abstract

Skeletal muscle can repair muscle tissue damage, but significant loss of muscle tissue or its long-lasting chronic degeneration makes injured skeletal muscle tissue difficult to restore. It has been demonstrated that non-thermal atmospheric pressure plasma (NTP) can be used in many biological areas including regenerative medicine. Therefore, we determined whether NTP, as a non-contact biological external stimulator that generates biological catalyzers, can induce regeneration of injured muscle without biomaterials. Treatment with NTP in the defected muscle of a Sprague Dawley (SD) rat increased the number of proliferating muscle cells 7 days after plasma treatment (dapt) and rapidly induced formation of muscle tissue and muscle cell differentiation at 14 dapt. In addition, *in vitro* experiments also showed that NTP could induce muscle cell proliferation and differentiation of human muscle cells. Taken together, our results demonstrated that NTP promotes restoration of muscle defects through control of cell proliferation and differentiation without biological or structural supporters, suggesting that NTP has the potential for use in muscle tissue engineering and regenerative therapies.

Muscle injury commonly has multiple causes, such as diseases, exposure to myotoxic agents, sharp or blunt trauma, ischemia, and exposure to extreme environments^1^. However, skeletal muscle has an amazing capacity to regenerate after injury through an elaborately orchestrated regenerative progress that take place at the tissue, cellular, and molecular levels^2^,^3^. This regenerative ability is mainly attributed to undifferentiated satellite cells because of their location at the periphery of mature skeletal myofibers. It is known that satellite cells are arrested at an early stage of the myogenic program. Under normal conditions, they are quiescent, but when injury occurs, satellite cells are activated, proliferated and differentiated to give rise to the muscle cells. The muscle cells then fuse with each other to form muscle fibers. Thus, satellite cells play an important role during skeletal muscle repair after injury. In addition, they can be renewed to support additional rounds of regeneration. Even though skeletal muscle has a strong ability to regenerate after injury, dynamic mechanisms of muscle regeneration are limited when a major loss of tissue occurs such as significant traumatic damage or long-lasting chronic degeneration^4^. To overcome these limitations, muscle regeneration requires exogenous factors for structural and functional recovery of large-scale muscle damage. As a strategy of regenerative medicine, it was reported that transplanting biomaterials and/or cells and treating biological factors achieved modest therapeutic effects^5^,^6^. In addition, it is well-known that external stimulation such as mechanical, electrical and laser stimulation plays an important role in muscle regeneration^7^,^8^,^9^.

Plasma is referred to as the fourth state of matter, after solids, liquids, and gases^10^. It was recently demonstrated that plasma can be used for a number of bio-medical applications. Thus, plasma medicine is emerging as a novel therapeutic technology in diverse bio-medical fields including blood coagulation, disinfection, control of inflammation, and regenerative medicine^11^,^12^. Specifically, non-thermal atmospheric pressure plasma (NTP) has been developed for application in the clinical field over the past few decades. In our previous study, we demonstrated that NTP could control the survival and phenotype of cells in a specific environment^13^,^14^,^15^. In addition, it was also reported that NTP induced wound healing, the differentiation of mesenchymal stem cells, and the development of limb buds, although the molecular mechanisms remained unclear^16^,^17^,^18^. However, therapeutic effect of NTP on muscle regeneration has not yet been determined.

In this study, we investigated for the first time whether NTP as a non-contact external stimulator could induce the natural regeneration of muscle defects without biological or structural support and evaluated the therapeutic effect of NTP on muscle regeneration *in vitro* and *in vivo*.

## Results

### Properties of N_2_-based non-thermal atmospheric pressure plasma

**Figure 1:**
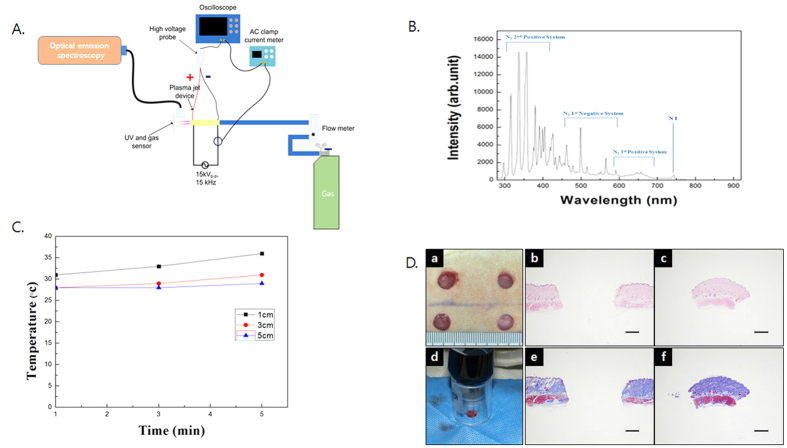
(**A**) Diagram of developed N_2_-based NTP (non-thermal atmospheric pressure plasma) system. (**B**) Spectrum analysis of N_2_-based NTP with optical emission spectroscope showed a variety of excited species in the plasma jet. (**C**) Temperature changes at various distances (1, 3, and 5 cm) were measured with a non-contact IR thermometer. The maximum temperature was 37 °C, similar to human body temperature at even 1 cm distance. (**D**) a: picture of muscle defects on a rat back. b and c: H & E staining of removed tissue. d: The NTP treatment was performed through an enclosed spacer to avoid NTP interference in the control defect site, and the non-treated site protected with surgical cloth during plasma treatment. e and f: Masson’s trichrome staining of removed tissue. Muscle tissue was detected in removed tissue, Scale bar = 2 mm.

A variety of excited species was found over a wide range of wavelengths from 280 nm to 920 nm. Nitrogen species including N_2_ second positive systems (290~410 nm), first positive systems (600~700 nm), and N_2_ first negative systems (410~600 nm) were revealed. Moreover, the oxygen ion (O_2_+) was measured at 500~600 nm (Fig. 1A,B). The temperature reached the saturation state in approximately 5 minutes, and the maximum temperature was 37 °C, similar to human body temperature at 1 cm distance (Fig. 1C). The temperature results indicated that the NTP jet could be safely applied to the tissue, and the NTP treatment was applied using a laboratory device and nozzle. The cell survival of the muscle cells was not affected by the NTP treatment (supplementary Fig. 2D).

### NTP restored defects by providing regenerated muscle tissue

**Figure 2:**
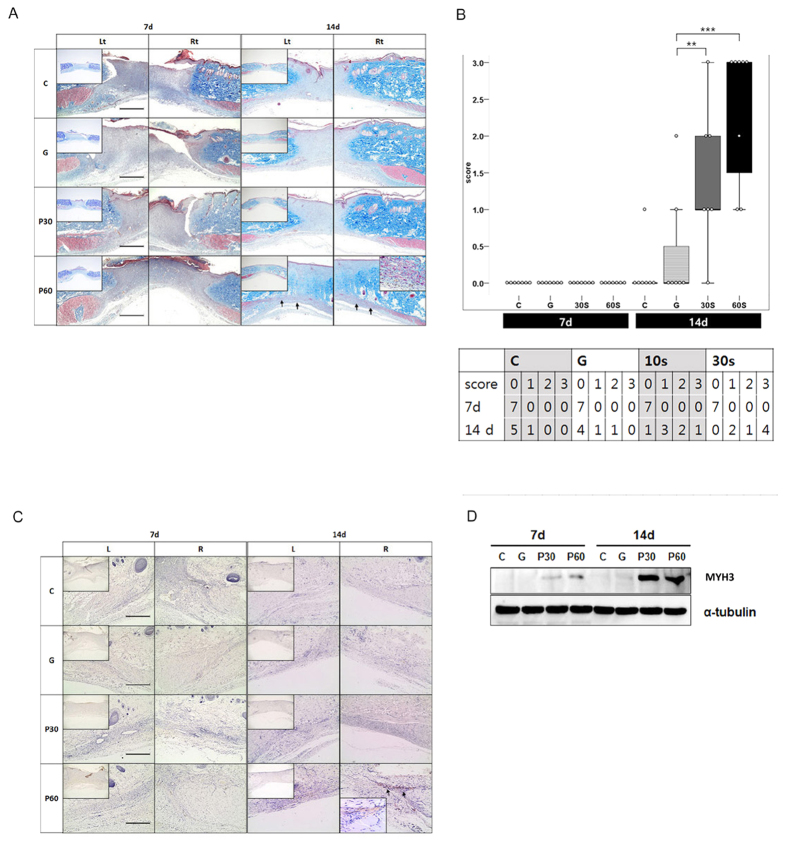
Evaluating tissue formation on muscle defect. (**A**) The Masson’s trichrome staining demonstrated regenerated muscle at the muscle defect site of the P 60 group at 14 days (black arrow), but it was not detected in the other groups. (**B**) Scoring of regenerated muscle. NTP (non-thermal atmospheric pressure plasma) treatment (P30 and P 60) accelerates muscle regeneration at day 14 (**P < 0.01, ***P < 0.001). (**C**) Expression of myosin heavy chain-3 (MYH3) in regenerated muscle tissues. Formation of new muscle was demonstrated by expression of MYH3 in the extended tissue from injured muscle fiber of the P 60 group at day 14 after NTP treatment (black arrow). (**D**) MYH3 expression was detected by western blot. All the Western blotting experiments were performed under the same condition. After transferring the blots onto PVDF membranes, we immediately cropped the targeted blots according to referenced indicating markers, and then targeted proteins were immunoblotted with its specific antibody and an α-tubulin antibody for normalization of protein. Scale bar = 1000 μm.

To test whether NTP affected muscle regeneration at the defect sites, we performed morphological, histological and immuno-histochemical analyses. On morphological examination, the tissues in all groups contained no traces of granulation tissue or inflammatory response. Micro-vessels were observed at the recovered sites in the P 60 group at 7 days after the NTP treatment (dapt). This phenomenon appeared markedly over time and was also observed in other groups at 14 dapt (supplementary Fig. 3, yellow arrow). On histological examination, regenerated muscle-like tissue was detected on the defect sites of the P 60 group at 14 dapt (Fig. 2A; black arrow), but it was not detected in the other groups. The regenerated muscle-like tissue scores for each group at 7 days showed no significant difference at 0 ± 0; the scores for each group at 14 days were as follows: C: 0.14 ± 0.38, G: 0.43 ± 0.78, P30: 0.85 ± 1.07, P60: 1.86 ± 1.21. The scores in the NTP-treated groups (P 30 and P 60) were higher than those of the controls at 14 days, and the result was statistically significant (Fig. 2B; P^**^ < 0.01, P*** < 0.001). To investigate the muscle characteristics of the regenerated tissue, we analyzed embryonic myosin heavy chain 3 (MYH3), which is regarded as a marker of muscle regeneration^8^. In immunohistochemical analysis to detect MYH3, MYH3 expression appeared on the extended tissue from injured muscle fibers in P 60 at 14 dapt (Fig. 2C). As a result of western blot examination, MYH3 levels were proportional to the immunohistochemical results. The MYH3 expression in the P 60 group was extremely high at 7 and 14 dapt (Fig. 2D).

### NTP enhanced muscle cell activation and muscle tissue formation

**Figure 3:**
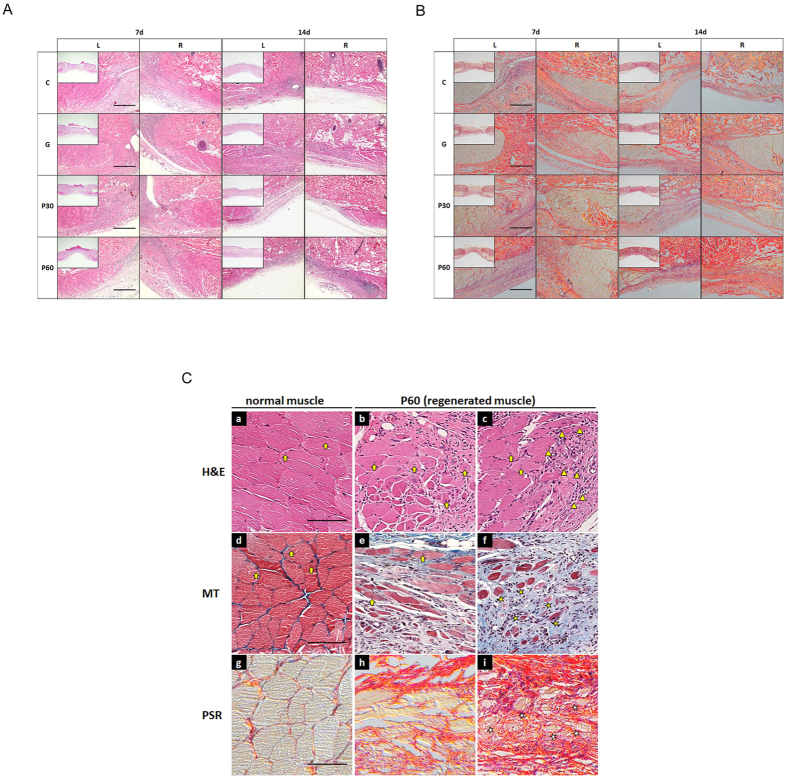
Analysis of cellular events induced by treatment with NTP (non-thermal atmospheric pressure plasma) at injured muscle tissues. (**A**) H & E staining and (**B**) PSR (picro Sirius red) staining were performed. (**C**) Observation of cellular events at higher magnification. The population of many cell clusters located at the adjacent regenerating myofibers (triangle). The small-caliber myofibers were detected far away from the injured muscle tissues (asterisk). The centrally localized myonuclei at newly formed myofibers were observed at the discrete segments of regenerating myofibers (arrow on b, d, and e). MT means Masson’s trichrome staining. Scale bar: A and B = 1000 μm, C = 100 μm.

**Figure 4:**
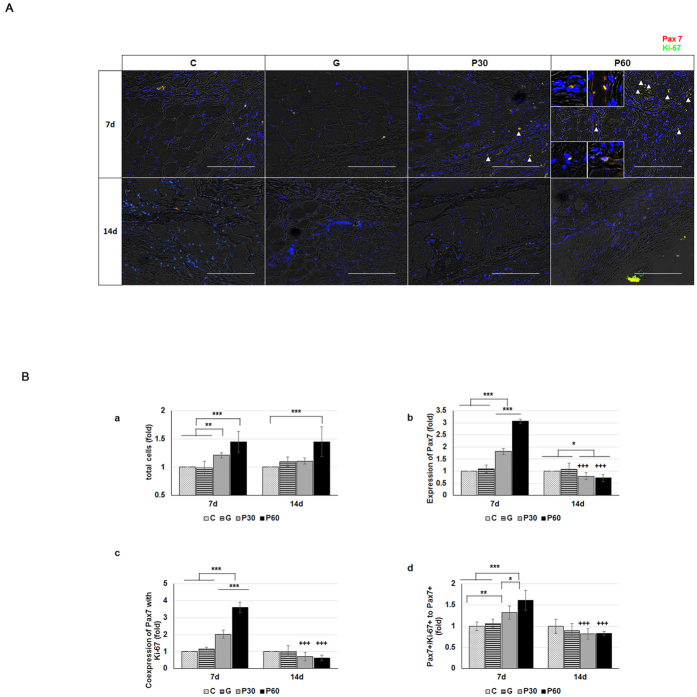
Analysis of satellite cell activation by NTP (non-thermal atmospheric pressure plasma). (**A**) Observation of proliferating satellite cell (Pax7+/Ki67+) at injured muscle tissues. Co-expressions of Pax7+/Ki67+ are seen as yellow spots. (**B**) Relative quantification of a: total cells. b: Pax7-expressed cells. c: co-expressed cells (Pax 7 and Ki-67). d: a ratio of Pax 7 to co-expressed (Pax7+/Ki-67+) cells. The values of each results showed that the P 30 and P 60 groups were higher than those of the control and gas-treated groups at day 7 (n = 7 per group). The asterisk (*) means significant differences in each group at the same time, and the plus (+) means significant differences of same group with time. *P < 0.05, **P < 0.01, and ***^,+++^P < 0.001. Scale bar = 200 μm.

To further examine the role of NTP in muscle cells and injured muscle tissue, we compared immunofluorescence-staining images from each group (Fig. 3A,B). In the tissue sections from P 60 at 7 and 14 dapt, high populations of cell clusters were detected near the regenerated myofiber (Fig. 3C; triangle). In the P 60 group, newly formed myofibers were clearly distinguished by elongated morphology at the border of injured muscle tissue of P 60 at 14 dapt (Fig. 3C; e,h). Distant from the injured muscle tissue, the small-caliber myofibers were found by unfusing them (Fig. 3C; f,I, asterisk). The centrally localized myonuclei on the newly formed myofibers were observed in discrete segments of regenerating myofiber (Fig. 3C; b,c,e; arrow) and were distinguished from peripherally located myonuclei on normal myofibers (Fig. 3C; a,d; arrow). To evaluate the characteristics of the cell clusters on the regenerating myofibers, we observed Pax7 and Ki-67 expression on the tissues (Fig. 4A). The numbers of total cells in the NTP-treated groups, P30 and P60, were 1.2 and 1.4 times higher than those in the control group at 7 dapt, respectively (Fig. 4B; a). There were no statistical differences over time. The numbers of Pax7+ cells in P30 and P60 were 1.8 and 3.1 times higher than in the control group, with statistical significance, at 7 dapt (Fig. 4B; b, P < 0.001). Over time, the cell counts in the NTP-treated groups were significantly lower. The nuclei that stained positively with Ki-67 on Pax7 (Pax7+/Ki-67+) were detected as proliferating satellite cells in the adjacent regenerating myofibers in P 30 and P 60 at 7 dapt but were scarcely visible at 14 dapt (Fig. 4A; yellow in small square, triangle). Moreover, quantitative analysis of the immunostaining showed that coexpression of Ki-67 with Pax 7 satellite cells in the NTP-treated groups (P30 and P60) was 2.0 and 3.6 times higher than they were in the control group at 7 days, respectively (Fig. 4B; c, P < 0.001). Similarly, Pax7+/Ki-67+ to Pax7 nuclei in P 30 and P 60 were 1.3 and 1.6 greater in number than in the control group at 7 days, respectively (Fig. 4B; d, P < 0.01 and P < 0.001). Over time, the nuclei counts in both P 30 and P 60 were significantly lower at 14 dapt. In each quantitative analysis, no statistical differences were observed between the control (C) and gas-treated (G) group, and the values in P 30 and P 60 were significantly reduced at 14 dapt on every quantitative analysis except for that of total cells. These results show that NTP treatment could increase the number of total cells, Pax7 expression and Pax7+/Ki-67+ (proliferating satellite cells) coexpression. We also determined whether Pax3+/Ki67+ double positive cell number increased in P30 and P60 group since Pax3 is also a well-known satellite cell marker. Supplementary Fig. 4 showed that P30 and P60 group have more Pax3+/Ki67+ double positive cells, which coincides with the result of Fig. 4A.

**Figure 5:**
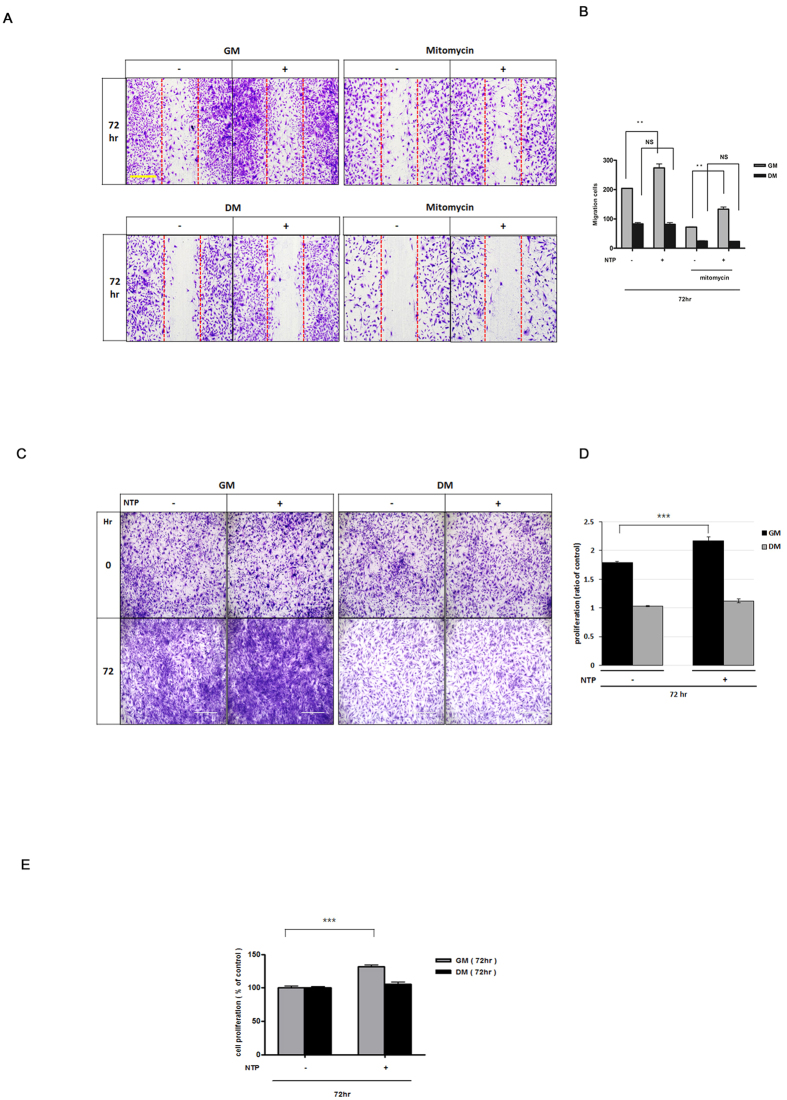
Evaluation of cell migration and proliferation on growth media (GM) and differentiation media (DM) *in vitro*. (**A**) Observation of cell migration *in vitro*. (**B**) The migrated area of the NTP (non-thermal atmospheric pressure plasma)-treated group was significantly higher than that of the control group in GM. The result of (**C**) crystal violet staining, and (**D**) the quantitative results of the cell density showed that treatment with NTP induced cell proliferation. (**E**) BrdU assay. NTP-treated cells were more proliferated than NTP-non treated cells in GM condition. ***P < 0.001. Scale bar = 1000 μm.

Next, to investigate whether the NTP treatment directly affected the muscle cells, we performed *in vitro* wound healing assays and crystal violet staining to evaluate cell migration and proliferation (Fig. 5). In the cell migration assay, the percentage of wound recovery area in the NTP-treated group (61.31 ± 6.3) was 1.5 times higher than the percentage in the control group (42.27 ± 4.13) with a statistical significance in the growth media (GM) condition at 72 hours. (Fig. 5A,B; P < 0.001). To determine the cell proliferation effect of NTP, we counted cells after crystal violet staining and compared the cell number to the initial seeded cell number. The result showed that the fold increase in the cell number of NTP-treated group (2.17 ± 0.01) was significantly higher than that of the control group (1.79 ± 0.02) in the GM condition (Fig. 5C,D; *P* < 0.001). In addition, we performed BrdU cell proliferation assay to confirm that the treatment with NTP could enhance muscle cell proliferation and the result showed that treatment with NTP could induce muscle cell proliferation (Fig. 5E). These *in vivo* and *in vitro* results indicate that the NTP treatment can induce muscle cell proliferation and migration.

### NTP induces muscle cell differentiation

**Figure 6:**
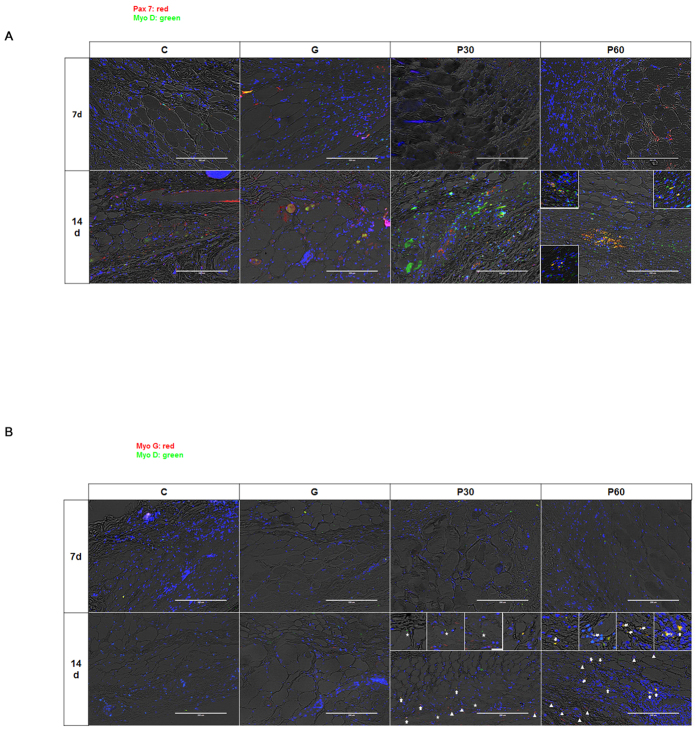
Analysis of muscle cell differentiation by NTP (non-thermal atmospheric pressure plasma). (**A**) Co-expression of Pax 7 with MyoD, which is regarded as a major event in muscle differentiation, was detected at the borders of the regenerating myofibers in the P 60 at day 14. (**B**) Co-expression of MyoD with myogenin (MyoG), which is regarded as a major event in the late stage of muscle differentiation, was detected in the regenerated tissues of the NTP-treated groups, P30 and P60 (white arrow). Expression of MyoD and MyoG is denoted by asterisks and triangles, respectively. Scale bar = 200 μm.

To examine the relevance of NTP treatment and muscle cell differentiation, we observed coexpression of Pax7/MyoD and MyoD/MyoG, which are regarded as major factors in muscle cell differentiation and late-stage markers of this differentiation in tissue. MyoD with Pax7 (Pax7 +/MyoD+) coexpression was detected on the borders of the regenerating myofibers in group P 60 at 14 dapt (Fig. 6A; yellow in small square). In the regenerated cell tissue in the NTP-treated groups (P30 and P60), MyoD +/MyoG+ coexpresion as a marker of late-stage muscle cell differentiation was also observed (Fig. 6B; white arrow).

**Figure 7:**
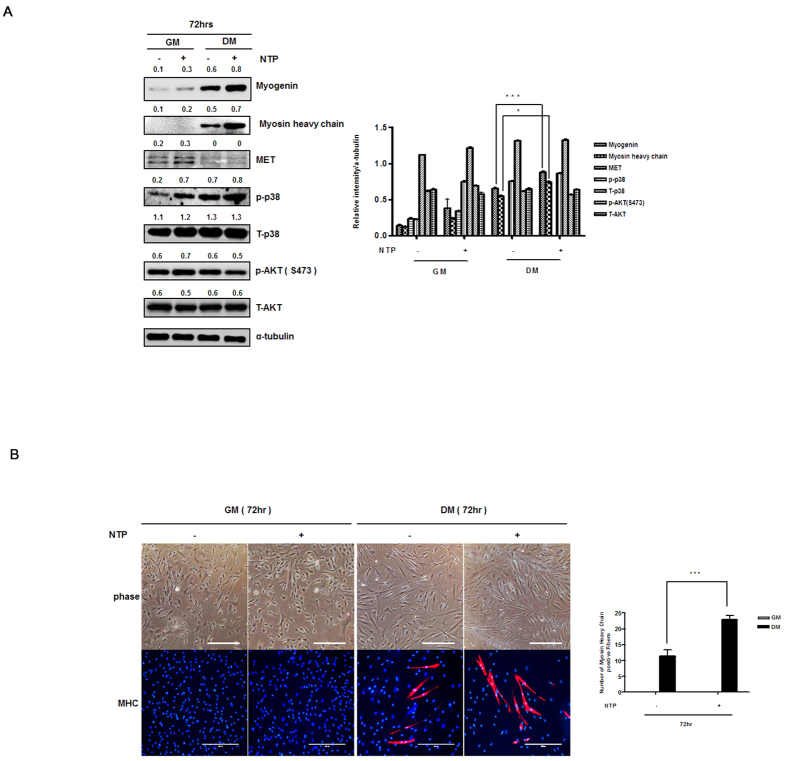
*In vitro* analysis of NTP (non-thermal atmospheric pressure plasma) effects on muscle cell differentiation. (**A**) Left: Western blot analysis. Expression of MHC, and myogenin in the differentiation media condition (DM) was higher with NTP treatment. All the Western blotting experiments were performed under the same condition. Cell lysates were prepared, run on a 10~12% SDS-PAGE gel, transferred to an PVDF membranes, we immediately cropped the targeted blots according to referenced indicating markers and an α-tubulin antibody for normalization of protein. Right: The band intensities were measured and represented as a graph. (**B**) Left: The myotube formation detected by staining of MHC in the DM condition was increased by NTP treatment. NTP treatment did not affect in GM. Right: MHC positive cells were counted and the positive cell number were represented as a graph. Scale bar = 200 μm.

To further examine the direct role of NTP in muscle cell differentiation, we observed the cell phenotypes and the expression of proteins and mRNAs known to be involved in myogenesis. When the myoblasts were cultured in DM, the cells were different; they became elongated and fused with nearby cells to form multinucleated tubes, and myogenin, myosin heavy chain, c-MET(HGFR), creatin kinase M, and p38 were significantly higher when muscle cell differentiation was initiated (supplementary Fig. 2). These results prompted us to examine the molecular mechanisms by which NTP induced muscle differentiation, and we asked which signaling mediators were regulated by NTP in muscle cell differentiation. Myosin heavy chanin and myogenin expression was increased in the NTP-treated cells, whereas the expression of MET was unchanged in the differentiation media condition. The intensity of the band was quantified and the values were presented as a graph (Fig. 7A). Finally, immune-fluorescence microscopy showed that the myotube formation with expression of MHC increased on the NTP-treated group at 72 hours and the number of MHC positive cells were described by a graph (Fig. 7B). These results indicate that treatment with NTP enhances muscle cell differentiation.

## Discussion

Skeletal muscles have the capacity to self-repair, but they cannot be restored after a major loss of tissue or in the event of long-lasting chronic degeneration^19^. As strategies for muscle regeneration in those environments, various materials and/or external stimulations have been used in attempts to restore injured muscle tissue^6^,^7^,^8^,^9^,^20^,^21^.

It has been demonstrated that NTP could be used as a novel treatment modality for a number of disorders. Our previous studies revealed that NTP has biological effects such as inhibiting cell invasion and inducing cancer cell death^14^,^15^. We also observed NTP’s reparative effects on a normal skin wound. In this study, we investigated whether NTP can be used for muscle regeneration in cases that are refractory and have poor prognosis. We evaluated whether NTP as a non-contact biological external stimulator for rapid muscle regeneration could induce the migration and proliferation of muscle cells *in vivo* and *in vitro* and restore muscle defects through newly formed myofibers without supporters such as biomaterials and biological factors.

Muscle regeneration studies have investigated a variety of muscles including murine tibialis anterior, the thigh, the gastrocnemius, and the rectus abdominus^8^,^21^,^22^,^23^. It is well-known that mechanical stimulation such as exercise induces muscle cell damage or regeneration^24^. In this investigation, we applied the panniculus carnosus muscle defect model because this muscle is influenced by compliant mechanical load and is readily accessible for experimental manipulation^25^.

The varying effects of NTP depend on a number of parameters such as the types of gases, defect size, the distance between the plasma nozzle and a target tissue, the pressure of gases for plasma generation, and the plasma treatment time. During pilot studies to optimize NTP application for muscle regeneration, we found that nitrogen gas-based NTP has excellent biological effects on muscle regeneration *in vitro* and *in vivo*. In terms of the defect size, smaller than 6 mm was unsuitable for this study because the skin layer was recovered too quickly to determine the effect of NTP on muscle regeneration (supplementary Fig. 3B). In contrast, the 8 mm muscle defect was still regenerating on day 30 (supplementary Fig. 3C). This result led us to use an 8 mm muscle defect for this study. The temperature was stable when the distance between the plasma nozzle and the target tissue was less than 5 cm and the gas releasing pressure was 10 (Fig. 1C). Thus, we used these conditions.

Our study was focused on the activation (proliferation and migration) and differentiation of satellite cells among the various cell types that are active in muscle regeneration because the roles of non-satellite cells for muscle regeneration are considered to be negligible compared with those of satellite cells^26^,^27^.

Further, both skeletal muscle homeostasis and muscle repair are mainly maintained by satellite cells. Satellite cells are juxtaposed to the myofiber, have low homeostatic turnover, and are maintained in a non-proliferative, quiescent state. Once they are activated, a process of self-renewal begins, or these cells may commit to migration into a injury site and differentiation for myofiber formation^28^,^29^; it is not, however, fully understood how the activated satellite cells select destination, self-renewal or myogenesis.

Satellite cells express a transcription factor, paired box 7 (Pax7), that is required for satellite cell proliferation. To determine the effect of NTP on the satellite cell proliferation, we counted the number of proliferating satellite cells, referred to as either myogenic precursor cells or myoblasts^30^,^31^. As shown in Fig. 4, the number of proliferating satellite cells, Pax7 positive and Ki67 positive, at the end of a strand of NTP-treated injured tissue was significantly higher than that in non-treated injured tissue (control group and gas group) at 7 dapt. The results of the *in vitro* studies also showed that the proliferation and migration of NTP-treated myoblast cells statistically increased in the GM condition. These results suggest that NTP activates satellite cells in muscle fibers and eventually allows them to initiate muscle proliferation.

The differentiation of activated satellite cells is also important during muscle regeneration^1^. The differentiation is controlled by the expression of myogenic differentiation 1 (MyoD), followed by the expression of myogenin (MyoG) in the late stage of differentiation^1^,^30^. We observed MyoD and MyoG expression on the borders of regenerating tissue in the NTP-treated groups at 14 dapt. The results were confirmed by showing that NTP induced the expression of a myosin heavy chain and myogenin in a human myoblast under culture with differentiation and even growth media *in vitro*. These results indicate that NTP can promote not only satellite cell proliferation but also the differentiation of the cells in injured muscle, implying that NTP induces satellite cell proliferation and drives the proliferating cells to enter a differentiation stage. Chernets *et al*. also suggested that NTP enhances a variety of biological activities such as survival, growth, and differentiation of cartilaginous elements within embryonic mouse autopods^18^. We cautiously suggest that NTP can affect some molecular-level need for proliferating muscle cells to differentiate and could convert into the initial differentiation state of muscle cells. Our recent data showed that NTP could activate STAT signaling in myoblast (unpublished data). It is widely accepted that myogenesis is critically dependent on STAT3, so it seems that NTP may enhance muscle cell differentiation through STAT3 activation. However, other numerous signaling pathways have also been found to influence myogenic differentiation. Thus, we are still investigating how NTP affect myogenic differentiation.

Taken together, we demonstrated for the first time that NTP could promote muscle regeneration without other factors, suggesting that NTP might be a good candidate as an external stimulator for muscle regeneration. Even though additional studies are necessary to determine whether NTP treatment combined with other biomaterials and/or biological factors, such as collagen, extracellular matrix, poly lactic-co-glycolic acid, IGF and VEGF, has a better effect on muscle regeneration, our results suggest that NTP alone is able to accelerate muscle repair after injury.

## Materials and Methods

### Physical properties of N_2_ based on non-thermal atmospheric pressure plasma

In this study, N_2_ gas as the source of plasma generation (Fig. 1A) was chosen based on our previous study that used a variety of gases (N_2_, Ar and He) (data not shown).

We carried out a spectrum analysis with an optical emission spectroscope (SV 2100, K-MAC, Korea) (Fig. 1B). Temperature changes at different distances were analyzed with a non-contact IR thermometer (Raytek, Santa Cruz, USA) on the N_2_ based NTP, which effused gas at 4 L/min (Fig. 1C).

### Overview of the animal study design

Animal care and procedures were in accordance with the National Institutes of Health Guidelines for the Care and Use of Laboratory Animals, and all experiments were approved by the Committee for Ethics in Animal Experiments of the Ajou University School of Medicine. All efforts were made to minimize animal suffering. Animals were cared for individually under isolated environmental conditions (pathogen free). Defects were induced in all animals, and each site was divided into 4 groups (n = 10) according to the plasma treatment or gas, namely: (I) defect as control (C), (II) N_2_ gas treatment (G), (III) plasma treatment for 30 seconds (P30), and (IV) plasma treatment for 60 seconds (P60). Each group (n = 10 from each group at each time point) was analyzed randomly at 7 days and 14 days post-injury. The animals in all groups remained healthy and survived the experimental period without disorder.

### Animal surgical procedures and NTP treatment

Twenty male Sprague Dawley rats (8 weeks; 250–300 g) were anesthetized with tiletamine (8.0 mg/kg; Virbac, Carros, France), zolazepam (8.0 mg/kg; Virbac, Carros, France) and xylazine hydrochloride (1.5 mg/kg; Bayer, Ansan, Korea). The distance between defects maintained a consistent gap of 20 mm to avoid biological interference with other defect sites. To induce the panniculus carnosus muscle defect on the same point of all rats, the coordinates for 4 defects were assigned based on the bones of the pelvic girdle. The defects were induced using a biopsy punch of 8 mm diameter (Miltex, York, USA) on the coordinates (Fig. 1D; a). The images of the induced defects were captured using a digital camera, and the defect sites in the captured images were automatically separated from the normal sites and calculated using Metamorph_NX image software (Molecular Devices, Sunnyvale, California, USA). The average diameter of twenty defects was 8.56 ± 0.47 mm. The removed tissues were stained to verify the consistency of the muscle defects in the rats, and the structures of the removed tissue contained muscle with the dermis layers (Fig. 1D; b,c,e,f). To avoid plasma interference and control the defect sites, the non-treatment sites were protected with surgical cloth during the plasma treatment. The treatment was confined to an enclosed space, and the distance between each defect and the plasma nozzle was constant at 40 mm by spacer (Fig. 1D; d).

### Histological evaluation and muscle regeneration scoring

At each time point, the site of interest in each sample was captured using a digital camera. The paraffin-embedded samples were sectioned serially from midline at a thickness of 4 μm and stained with hematoxylin-eosin (H&E) and Masson’s trichrome (MT) as was done previously^32^. MT staining was applied to analyze the muscle regeneration scores at each defect site. Five slides from each group (n = 7 from each group at each time point) were chosen randomly and captured using the EVOS FL auto cell imaging system (Thermo Fisher Scientific, Waltham, USA). The muscle elongation (muscle regeneration from the edge of the defect) was measured in Metamorph® NX image software and scored on the basis of our standard scoring system and standard sample (Supplementary Fig. 1)^33^.

### Immuno-histochemical and immuno-cytochemical analysis

Immuno-histochemical and immune-cytochemical analysis were performed as described previously^13^,^34^. The sections and cultured cells were incubated with antibodies, namely, anti-Pax7, anti-MyoD, anti-Myogenin (Abcam, Cambridge, UK), anti-Ki67, and anti-myosin heavy chain 3 (Santa Cruz, California, USA) antibodies to detect activated satellite cells, proliferating satellite cells, myogenic differentiation, and terminal differentiation^8^,^28^. Anti-rabbit alexa 488 and anti-mouse Cy3 antibodies (Thermo Fisher Scientific, IL) were used as secondary antibodies.

### Cell culture and differentiation

Primary normal human skeletal muscle myoblasts were obtained from Lonza and cultured to proliferate and differentiate following the method that was described in a previous manuscript. Undifferentiated cells in Clonetics Basal Medium and Bullet Kit medium were induced to differentiate by switching the medium to Dulbecco’s modified essential medium supplemented with 2% horse serum, 2 mmol/L glutamine, and 50 U/mL streptomycin and penicillin. The cells’ differentiation capacities were previously evaluated by expression of myogenic differentiation markers (Supplementary Fig. 2A–C).

### Cell migration, proliferation, and survival analysis

Cells were seeded in culture plates at a density of approximately 1.25 × 10^5^ cells/cm^2^ and grown to confluence. Cell migration assays and cell proliferation assays were performed as described previously^13^. For the quantitative analysis, each cell on a plate (n = 6) was captured with a digital imaging camera at each time point. The wound on the captured image was automatically recognized and measured by Metamorph® NX image software and eluate of crystal violet staining was measured on a spectrum of 540nm. LIVE/DEAD Viability/Cytotoxicity using calcein acetoxymethyl ester (Calcein-AM) and ethidium homodimer-1 (EthD-1) (Invitrogen, Carlsbad, USA) for the cell survival analysis were performed as described previously^35^. Fluorescence images were captured on the EVOS FL auto cell imaging system.

### Western blot and reverse transcription-polymerase chain reaction (RT-PCR)

Immunoblot and RT-PCR were performed as described previously^13^. The following antibodies were used for the western blot analysis: myosin heavy chain (R&D system, Minneapolis, USA), myogenin (Abcam, Cambridge, UK), creatin kinase M (Santa Cruz, California, USA), MET, p-p38, p38, p-AKT, AKT, and α-tubulin (Cell Signaling Technology, Danvers, Massachusetts, USA). PCR condition is as follow: denaturation for 3 min at 94 °C, amplification for 35 cycles of 30 s at 94 °C, 60.7 °C, and 72 °C, final extension for 5 min at 72 °C. The human myogenin primer sequences were: F, 5-AGC GCC CCC TCG TGT ATG-3; R, 5-TGT CCC CGG CAA CTT CAG C-3.

### Statistical analysis

All of the data were derived from independent experiments, and the parameters are expressed as the means ± standard deviations. The means for the different groups were compared using one-way analysis of variance and Tukey’s post hoc test. Statistical significance was set as p value less than 0.05 (*), 0.01 (**) and 0.001 (***).

## Additional Information

**How to cite this article**: Choi, J. W. *et al*. Novel Therapeutic Effects of Non-thermal atmospheric pressure plasma for Muscle Regeneration and Differentiation. *Sci. Rep.* **6**, 28829; doi: 10.1038/srep28829 (2016).

## Supplementary Material

Supplementary Information
