# Supplementary material for: Novel Therapeutic Effects of Non-thermal atmospheric pressure plasma for Muscle Regeneration and Differentiation
Source: Sci Rep. 2016 Jun 28;6:28829. doi: 10.1038/srep28829 (PMC4923893; doi:10.1038/srep28829)
Supplement: Supplementary Information [file srep28829-s1.pdf]

Novel Therapeutic Effects of Non-thermal atmospheric pressure plasma for Muscle Regeneration and Differentiation

Jae Won Choi, Sung Un Kang, Yang Eun Kim, Ju Kyeong Park, Sang Sik Yang, Yeon Soo Kim, Yun Sang Lee, Yuijina Lee, Chul-Ho Kim

Supplementary Figure 1.

A.

score	descriptions
0	No muscle expansion from defect
1	<25 % muscle expansion from defect
2	25 %≤ between <50 % muscle expansion from defect
3	<50 % muscle expansion from defect

B.

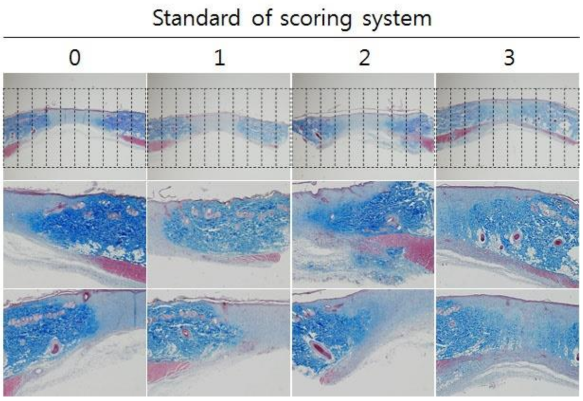

Supplementary Figure 1. Semi-quantitative analysis of regenerated muscle tissue. (A) Establishing standard scoring system. The scoring system was established as four grades based on expansion distance of muscles from defects. (B) Standard images for scoring. The Masson's trichrome staining of each group was semi-quantified based on our standard index and images. The distance of the elongated muscles from initial defects was converted to percent (%)

Supplementary Figure 2.

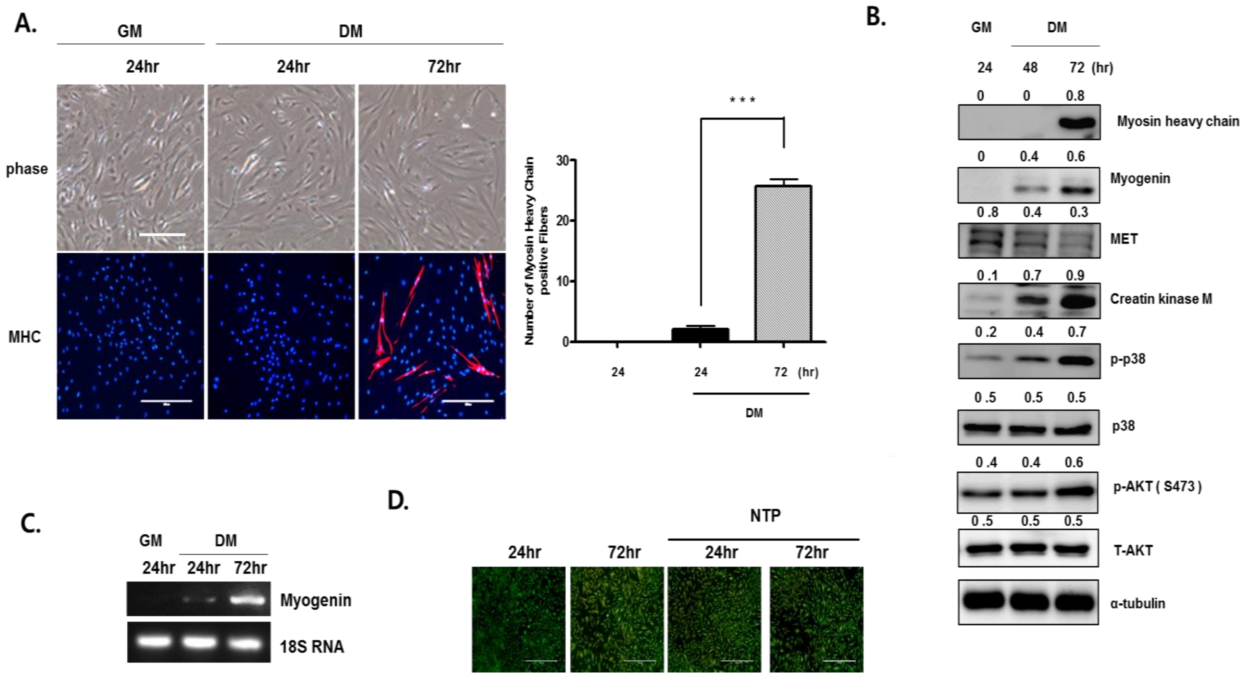

Supplementary Figure 2. Verification of primary human myoblast characteristics and validation of NTP (non-thermal atmospheric pressure plasma) cytotoxicity. (A) Morphology change of myotube formation with expression of myosin heavy-chain (MHC). (B) Protein expression of MHC, myogenin (MyoG), c-MET, creatine kinase M, and p38. (C) Gene expression of MyoG in myoblast in the DM condition. (D) Cell survival and cytotoxicity in muscle cells by NTP. Cytotoxicity by our NTP system was not observed and survival of muscle cells by NTP treatment was maintained at day 3. Scale bar; A = 200 μm, D = 1000 μm

Supplementary Figure 3

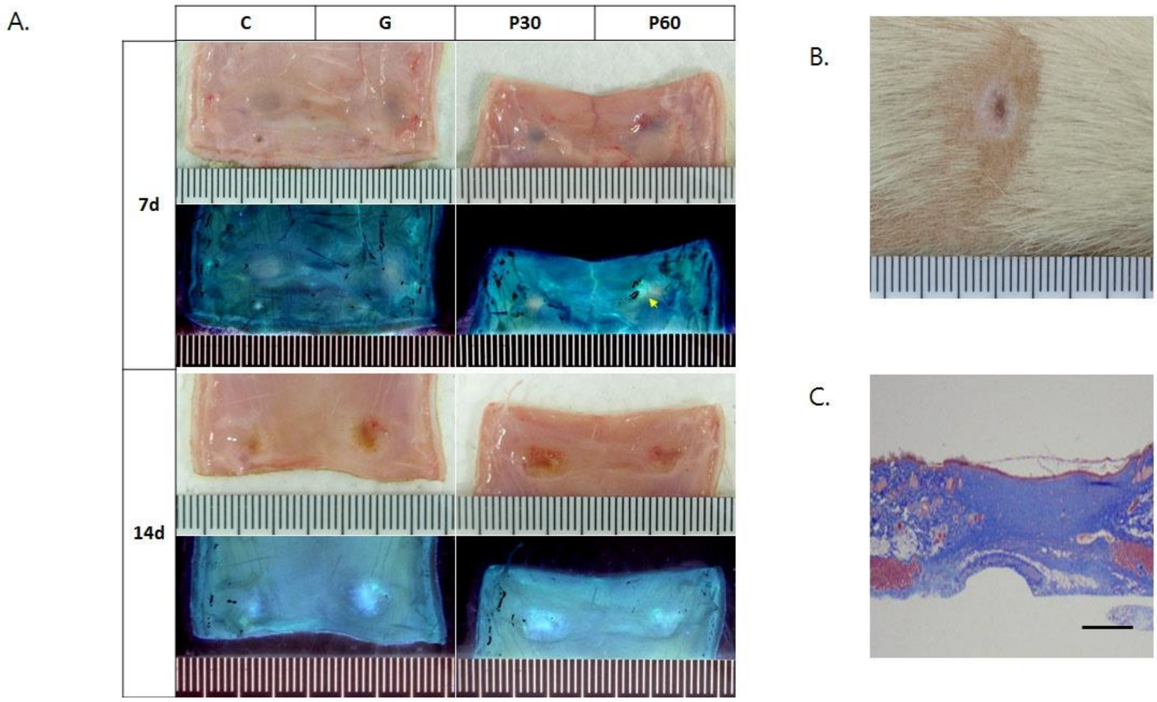

Supplementary Figure 3. Morphological and histological examinations at each time point. (A) Interesting sites of each sample were captured, and the images were converted using Metamorph® NX imaging software. (B) Rat skin was enclosed at day 11 when the 6 mm defect in the diameter of an acute full thickness rat muscle injury model was induced. (C) Rat skin was enclosed at day 30 when 8 mm muscle defect was induced, but muscle tissue was not naturally recovered. Scale bar = 1000 μm.

Supplementary Figure 4

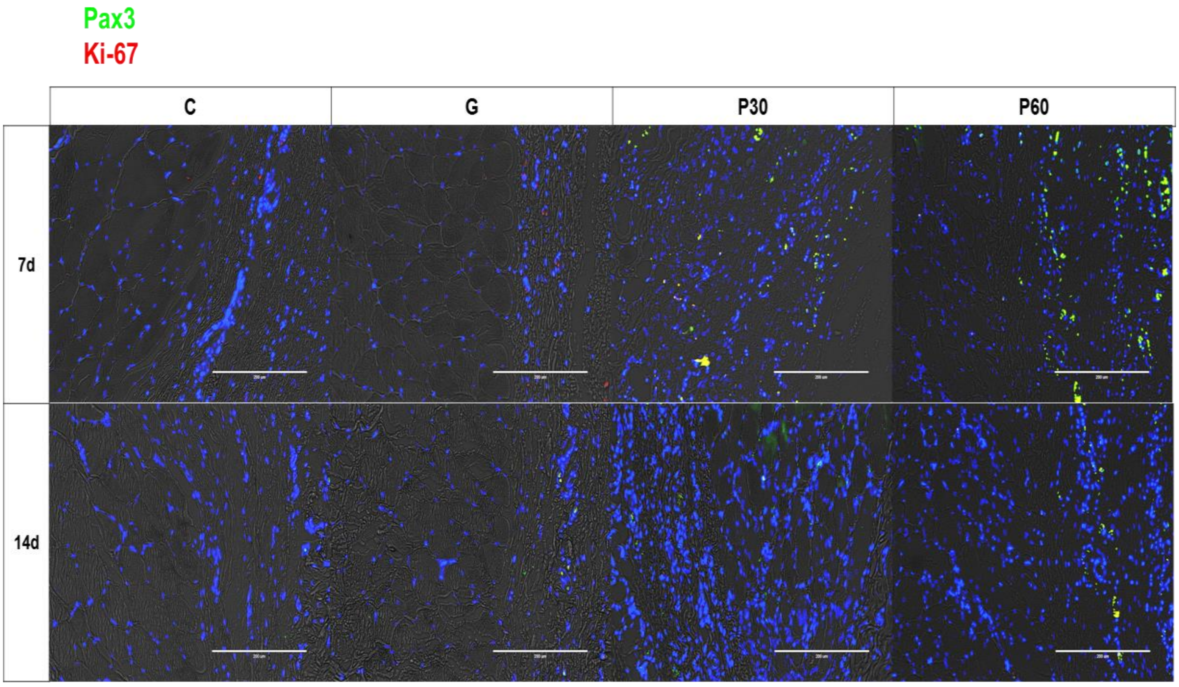

Supplementary Figure 4. Analysis of satellite cell activation by NTP (non-thermal atmospheric pressure plasma). Observation of proliferating satellite cell (Pax3+/Ki67+) at injured muscle tissues. Co-expressions of Pax3+/Ki67+ are seen as yellow spots.
